# 

**Published:** 1859-01

**Authors:** 


### CONTENTS.

### ORIGINAL COMMUNICATIONS.

PAGE

ARTICLE I.—Nitrogen, or Azote. By Prof. N. R. Wright, . 3

ARTICLE II.—A Brief Review of Dental Associations. By A Senior, . . . . . . . 16

ARTICLE III.—Preserving and Preparing Anatomical Material. By W. G. Smull, M. D. . . . . . 29

ARTICLE IV.—Mucous Polypi of the Maxillary Sinus. Translated for this Journal, . . . . . . 32

ARTICLE V.—Letter from M. Heyfelder on Disarticulation of the Lower Jaw. Translated for this Journal. . . 34

ARTICLE VI.—Resection of the Inferior Maxillary in a case of Cancer of the Bone. Translated for this Journal, . 36

ARTICLE VII.—Cancerous Tumor in the Right Parotid Region. Translated for this Journal. . . . 38

ARTICLE VIII.—Surgical Application of Galvanism. By Dr. Brown Sequard. Translated for this Journal, . . 40

ARTICLE IX.—Dentistry among the Ancients - . 43

ARTICLE XI.—Cheoplastic Method of Mounting Artificial Teeth, 57

### PROCEEDINGS OF SOCIETIES.

ARTICLE XII.—Meeting of the Western Dental Society, . 58

ARTICLE XIII.—Monthly Meeting of the College of Dentists, London, . . . . . . . 63

PAGE

### SELECTED ARTICLES.

ARTICLE XIV.—A Lecture on the Principles and Practice of Dental Surgery. By Alfred Carpenter, M. B., M. R. C. S., &c. . . . . . . . 109
ARTICLE XV.—On Artificial Dentures. By Dr. J. Allen, 115
ARTICLE XVI.—Lateral Pressure of the Teeth considered in relation to obscure Nervous Affections. By J. M'Calla, D. D. S., Lancaster, Pa. . . . . . 124

### EDITORIAL DEPARTMENT.

Nitric Acid and Caries, . . . . . . 141
The Dental Register of the West, . . . . . 147
American Dental Covention, . . . . . . 148
To Correspondents, . . . . . . . 149
Professional Items, . . . . . . . 150
OBITUARY.—Dr. Elisha Townsend, . . . . . 151

